# Effects of methamphetamine-induced neurotoxicity on striatal long-term potentiation

**DOI:** 10.1007/s00213-021-06055-8

**Published:** 2022-01-05

**Authors:** Anne S. Gibson, Peter J. West, Kristen A. Keefe

**Affiliations:** 1 Interdepartmental Program in Neuroscience, University of Utah, Salt Lake City, UT USA; 2 Department of Pharmacology and Toxicology, College of Pharmacy, University of Utah, 30 S 2000 E Rm 201, Salt Lake City, UT 84112 USA; 3 Anticonvulsant Drug Development Program, University of Utah, Salt Lake City, UT USA

**Keywords:** Methamphetamine, Neurotoxicity, Dopamine, Long-term potentiation

## Abstract

**Rationale:**

Methamphetamine (METH) exposure is associated with damage to central monoamine systems, particularly dopamine signaling. Rodent models of such damage have revealed a decrease in the amplitude of phasic dopamine signals and significant striatal dysfunction, including changes in the molecular, system, and behavioral functions of the striatum. Dopamine signaling through D1 receptors promotes corticostriatal long-term potentiation (LTP), a critical substrate of these striatal functions.

**Objectives:**

Therefore, the purpose of this study was to determine if METH-induced dopamine neurotoxicity would impair D1 receptor-dependent striatal LTP in mice.

**Methods:**

Mice were treated with a METH binge regimen (4 × 10 mg/kg *d,l*-methamphetamine, s.c.) that recapitulates all of the known METH-induced neurotoxic effects observed in humans, including dopamine toxicity. Three weeks later, acute brain slices containing either the dorsomedial striatum (DMS) or dorsolateral striatum (DLS) were prepared, and plasticity was assessed using white matter, high-frequency stimulation (HFS), and striatal extracellular electrophysiology.

**Results:**

Under these conditions, LTP was induced in brain slices containing the DMS from saline-pretreated mice, but not mice with METH-induced neurotoxicity. Furthermore, the LTP observed in DMS slices from saline-pretreated mice was blocked by the dopamine D1 receptor antagonist SCH23390, indicating that this LTP is dopamine D1 receptor-dependent. Finally, acute in vivo treatment of METH-pretreated mice with bupropion (50 mg/kg, i.p.) promoted LTP in DMS slices.

**Conclusions:**

Together, these studies demonstrate that METH-induced neurotoxicity impairs dopamine D1 receptor-dependent LTP within the DMS and that the FDA-approved drug bupropion restores induction of striatal LTP in mice with METH-induced dopamine neurotoxicity.

## Introduction

Methamphetamine (METH) is a highly addictive psychostimulant with negative health, social, and economic consequences. Treatments for METH use disorder are currently limited to behavioral therapies that are generally unsuccessful. Consequently, the National Institute on Drug Abuse has declared research to identify medications to treat METH use disorder a priority (NIDA 2020). Cognitive deficits are apparent in individuals with a history of METH abuse, and targeting cognitive function may be an efficacious approach to managing METH use disorder. However, to target cognitive dysfunction associated with METH use and to establish more successful treatments for METH use disorder, the neurotoxic effects of METH must be better understood.

The striatum is critically involved in cognitive processes such as learning, motivation, and behavioral flexibility, and striatal dysfunction can have significant cognitive and behavioral consequences. Studies using a METH binge regimen to model the long-term effects of METH-induced neurotoxicity report lasting changes to molecular, system, and behavioral functions of the striatum. Importantly, METH damages nigrostriatal dopamine neuron terminals, effectively inducing a partial dopamine lesion within the striatum (Kogan et al. 1976; Wagner et al. 1980; Grace et al. 2010; Ares-Santos et al. 2014). This lesion is apparent as reductions in striatal dopamine tissue content, evoked dopamine release, and dopamine uptake (Cass and Manning 1999; Howard et al. 2011). Furthermore, METH-induced neurotoxicity is associated with decreased amplitude and frequency of phasic dopamine signals within the striatum (Howard et al. 2011, 2013; Robinson et al. 2014). Together, these studies demonstrate that the striatal dopamine system, particularly phasic dopamine signaling, is significantly impaired within the context of METH-induced neurotoxicity.

In addition to the deficits in dopamine signaling, METH-induced neurotoxicity is also associated with impairments in striatally mediated behaviors that involve dopamine signaling. For example, rats with METH-induced neurotoxicity show cognitive impairments during striatally based egocentric maze tasks (Chapman et al. 2001; Daberkow et al. 2005; Herring et al. 2008; Vorhees et al. 2011; Gutierrez et al. 2018) and impairments during habitual learning (Son et al. 2011). Given that phasic dopamine signaling modulates synaptic plasticity related to reward learning (Reynolds et al. 2001) and is sufficient to form associations underlying striatally based learning (Tsai et al. 2009; Zweifel et al. 2009; Steinberg et al. 2013), impairments in phasic dopamine signaling as a consequence of METH-induced toxicity provide a possible explanatory link between the partial dopamine loss induced by METH and the impairments in striatally based learning and memory.

Long-term synaptic plasticity is an important component of normal striatal function and is a neural correlate of learning and memory. Thus, one possible mechanism through which impairments in phasic dopamine signaling may affect learning and memory, and consequently behavior, is through altering corticostriatal synaptic plasticity, particularly long-term potentiation (LTP) (Calabresi et al. 1992). Medium spiny neurons (MSNs) make up approximately 90–95% of the cells within the striatum and receive dopaminergic input from the substantia nigra and glutamatergic input from the cortex and thalamus (Gerfen 1992). MSNs generally selectively express either dopamine D1 receptors or D2 receptors, and are referred to as D1-MSNs or D2-MSNs, respectively (Gerfen et al. 1990). Through these receptors, dopamine acts as a bidirectional modulator of corticostriatal long-term plasticity. In general, dopamine D1 receptor signaling promotes LTP, whereas dopamine D2 receptor signaling promotes long-term depression (LTD) (Lovinger 2010).

In summary, dopamine is a critical modulator of striatally based behaviors, and dopamine D1 receptor signaling promotes striatal LTP. METH is associated with a number of neurotoxic consequences, including deficits in striatally mediated learning and memory and damage to the striatal dopamine system. Therefore, the purpose of this study was to determine if corticostriatal LTP is also impaired within the context of METH-induced neurotoxicity. The non-contingent METH binge regimen was used to induce METH neurotoxicity, and corticostriatal LTP was assessed in acute brain slices approximately 3–5 weeks after METH exposure. Furthermore, we examined whether bupropion, a clinically used medication, could reverse the discovered METH-induced changes in LTP as a first step to assessing its potential efficacy for managing corticostriatal deficits associated with METH-induced neurotoxicity.

## Materials and methods

### Animals

Adult male C57BL/6 J mice (The Jackson Laboratory, Bar Harbor, ME; 2–3 months old) were group-housed on a 12:12-h light cycle. Animal care and experimental procedures followed the *Guide for the Care and Use of Laboratory Animals* (8^th^ Ed.) and were approved by the Institutional Animal Care and Use Committee at the University of Utah.

### Methamphetamine pretreatment

Mice were rehoused 8/cage and treated with a neurotoxic METH binge regimen. Briefly, mice received one injection of either METH (*d,l*-methamphetamine hydrochloride, 10 mg/kg calculated as the free base, s.c.; NIDA Drug Supply Program, Research Triangle Park, NC) or saline (10 mL/kg) once every 2 h for a total of four injections over eight hours. On alternating hours, body temperature was recorded using a rectal probe. However, a portion of mice used in the bupropion LTP experiment did not have their body temperature monitored due to COVID-19 distancing restrictions. The day after the METH binge regimen, mice were returned to their home cages and allowed to recover for a minimum of 3 weeks before being used for electrophysiology experiments.

### Acute bupropion treatment

METH-pretreated mice used for the bupropion LTP experiment were injected with bupropion hydrochloride (25 mg/kg or 50 mg/kg calculated as the free base, i.p.; Sigma-Aldrich, St. Louis MO) or saline vehicle (10 mL/kg) and then sacrificed 30 min later, as described below.

### Acute sagittal slice preparation

Mice were deeply anesthetized with isoflurane and decapitated. The brain was rapidly removed and temporarily submerged in partially frozen, oxygenated (95% O_2_/5% CO_2_) sucrose cutting solution (180 mM sucrose, 3 mM KCl, 26 mM NaHCO_3_, 1.4 mM NaH_2_PO_4_, 10 mM glucose, 0.5 mM CaCl_2_, 3 mM Mg_2_SO_4_). The brain was then cut down the midline, and the hemispheres were glued lateral side down to Leica VT1000S specimen disc. In some cases, only the left hemisphere was used for electrophysiology, and the right hemisphere was saved for later determination of dopamine toxicity via dopamine transporter (DAT) immunohistochemistry (IHC). The brain was then submerged in partially frozen, oxygenated sucrose cutting solution, and 300-μm sagittal slices were cut using a Leica VT1000S vibrating microtome. The first two slices containing the striatum were designated DMS and consisted of striatum from approximately 1 to 1.6 mm lateral to midline. The last two slices containing the striatum were designated DLS and consisted of striatum from approximately 2.4 to 3 mm lateral to midline. All slices were collected and placed in a recovery chamber filled with oxygenated artificial cerebral spinal fluid (ACSF; 126 mM NaCl, 3 mM KCl, 26 mM NaHCO_3_, 1.4 mM NaH_2_PO_4_, 10 mM glucose, 2.5 mM CaCl_2_, 1 mM Mg_2_SO_4_, 290–305 mOsm, 7.3–7.4 pH) for 1 h.

### Electrophysiology

Recordings were performed on an 8 channel Scientifica SliceMaster electrophysiology workstation that allows an experimenter to independently record field excitatory postsynaptic potentials (fEPSPs) from multiple slices concurrently. Slices were transferred to Slicemate recording chambers and continuously perfused with oxygenated ACSF (2.5 mL/min, 30–31 °C) or, when designated, ACSF + 10 μM SCH23390, a dopamine D1 receptor antagonist. A twisted nichrome/formvar wire bipolar stimulating electrode was placed at the interface of the corpus callosum and striatal white matter tracts. A borosilicate glass recording electrode (2–4 MΩ) was placed in the adjacent gray matter of the striatum. fEPSPs were evoked every 30 s with 100-μs duration constant-voltage stimuli using Slice‐ISO stimulators (npi electronic GmbH, Tamm, Germany); stimulation intensity was sufficient to evoke a half-maximal fEPSP responses. Baseline fEPSPs were recorded for 30 min, followed by 1 s of high-frequency stimulation (HFS, 100 Hz, designated as time 0 in graphs), and then 1 h of post-HFS recording with fEPSPs evoked every 30 s.

Data were sampled at 10 kHz, with a low-pass filter set to 1 kHz, a high-pass filter set to 3 Hz, and a gain of 500. For each slice, the amplitudes of the CNQX-sensitive component of the last five fEPSPs before HFS were averaged and set to 100%, as previously described in Nagarajan et al. (2017). All fEPSP amplitudes were expressed as a percent of this baseline. All data from a slice recording were excluded if any of the following exclusion criteria were met: (1) average baseline change of greater than 20%, (2) baseline fEPSP < 0.2 mV.

### Tissue sectioning and immunohistochemistry

Following sacrifice, the right hemispheres from all of the METH-pretreated mice and a portion of the saline-pretreated mice were collected and used for DAT IHC to assess METH-induced dopamine toxicity. The right hemisphere was fixed in 4% formaldehyde in 0.1 M PBS at 4 °C for 24 h, cryoprotected in 30% sucrose in 0.1 M PBS at 4 °C, and then flash-frozen in isopentane. Brains were cryosectioned into 30-μm coronal slices containing the striatum and stored at 4 °C in 0.02% sodium azide in 0.1 M PBS until use. DAT IHC was done on the free-floating sections using a rat anti-dopamine transporter monoclonal antibody (Millipore #MAB369), biotinylated rabbit anti-rat IgG secondary antibody (Vector #BA-4001), ABC peroxidase kit (Vector #PK-6100), and DAB-NiCl (Vector #SK-4100) substrate kit. Images were captured on a lightbox, digitized, and analyzed using ImageJ software. Densitometric analysis was completed by measuring background-subtracted average gray values in the DMS and DLS and expressing these values as a percent of saline controls.

### Statistical analysis

For electrophysiology experiments, sample sizes are expressed in figures as *n* = slice number/*n* = animal number. Analyses were done using the slice sample size, GraphPad Prism 7.0a software, and JMP Pro 14 software. To determine group differences for electrophysiology experiments, average fEPSP amplitudes during the last 10 min of recording were calculated for each slice, grouped, and analyzed with two-way repeated measures ANOVAs and post hoc Sidak’s multiple comparisons tests, or a repeated measures MANOVA with a post hoc Dunnett’s multiple comparison test as necessary. To determine LTP or LTD expression, average fEPSP amplitudes during the last 10 min of recording were grouped and analyzed with a one-sample *t*-test comparison to a theoretical value of 100%. LTP was defined as a group average statistically above a theoretical value of 100%. LTD was defined as a group average significantly below a theoretical value of 100%. Group differences in body temperature were analyzed using a two-way repeated measures ANOVA and post hoc Sidak’s multiple comparisons test. DAT IHC images were analyzed using unpaired *t*-tests or a one-way ANOVA as necessary. Data are represented as mean ± *SEM*, and significance was set for all analyses at *p* < 0.05.

## Results

### METH elevates body temperature and damages dopamine innervation of the striatum

**Fig. 1:**
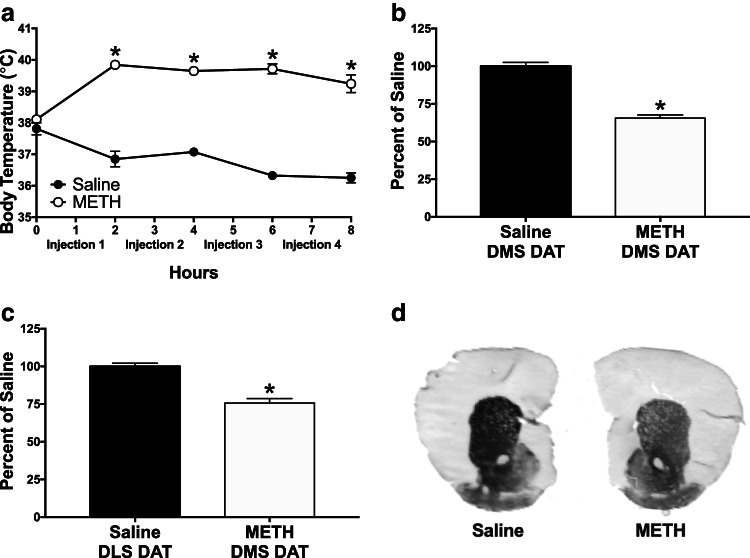
METH elevates body temperature and damages dopamine terminals. **a** The METH binge regimen elevates body temperatures compared to saline controls. Rectal temperature was taken at hours 0, 2, 4, 6, and 8. METH or saline injections were given at hours 1, 3, 5, and 7. A two-way repeated measures ANOVA of body temperature revealed a significant main effect of treatment (*F*_(1, 33)_ = 172.2, *p* < 0.0001), a significant main effect of time (*F*_(4, 132)_ = 2.972, *p* = 0.0218), and a significant treatment × time interaction (*F*_(4, 132)_ = 16.24, *p* < 0.0001). A post hoc Sidak’s multiple comparisons test found that the METH binge regimen (*n* = 27) induced significant hyperthermia compared to saline controls (*n* = 8) at all time points after the first dose of METH. **b** The METH binge regimen (*n* = 12) reduces immunohistochemical staining of DAT in the DMS. Data are expressed as a percent of the average values from the saline-pretreated controls (*n* = 7). **c** The METH binge regimen (*n* = 9) reduces immunohistochemical staining of DAT in the DLS. Data are expressed as a percent of the average values from the saline-pretreated controls (*n* = 9). **d** Representative immunohistochemical staining of DAT in coronal brain slices containing the DMS and DLS from saline-pretreated and METH-pretreated mice. All data are expressed as group mean ± *SEM*. *Significantly different from saline group, *p* < 0.05

The METH binge regimen, consisting of four non-contingent, subcutaneous injections of 10 mg/kg METH, reliably induces all of the known neurotoxicity observed in human METH abuse, including dopamine toxicity. Hyperthermia is an important contributor to this METH-induced dopamine toxicity (Albers et al. 1995). During the METH binge regimen, a two-way repeated measures ANOVA of body temperature revealed a significant main effect of treatment (Fig. 1a, F_(1, 33)_ = 172.2, *p* < 0.0001), a significant main effect of time (*F*_(4, 132)_ = 2.972, *p* = 0.0218), and a significant treatment × time interaction (*F*_(4, 132)_ = 16.24, *p* < 0.0001). A post hoc Sidak’s multiple comparisons test found that the METH binge regimen induced significant hyperthermia compared to saline controls at all time points after the first dose of METH, with the METH group (*n* = 27) reaching a maximum body temperature of 39.8 ± 0.1 °C versus 37.8 ± 0.2 °C for the saline group (*n* = 8).

The DAT is present on dopamine terminals, and drugs that induce dopamine neurotoxicity, such as METH, decrease DAT expression (Friend et al. 2013; Son et al. 2013; Fricks-Gleason et al. 2016). Densitometric analysis of DAT immunohistochemistry within the DMS and DLS was therefore used to confirm dopamine neurotoxicity resulting from the METH binge regimen. The METH binge regimen led to a significant reduction in immunohistochemical staining of DAT in both the DMS (Fig. 1b, Saline DMS: 100.0 ± 2.5, *n* = 7; METH DMS: 65.6 ± 2.1, *n* = 12; *t* = 10.34, *df* = 17, *p* < 0.0001) and DLS (Fig. 1c, Saline DLS: 100.0 ± 2.2, *n* = 9; METH DLS: 75.6 ± 3.0, *n* = 9; *t* = 6.625, *df* = 16, *p* < 0.0001), when compared with unpaired *t*-tests to expression in saline controls. Thus, the METH binge regimen induced lasting dopamine neurotoxicity in both the DMS and DLS. Furthermore, in the bupropion LTP experiments where only METH-pretreated groups were used, a one-way ANOVA revealed that there was not a significant difference in DAT expression between groups (DMS: *F*_(2, 28)_ = 1.173, *p* = 0.3241, data not shown; DLS: *F*_(2, 23)_ = 0.6379, *p* = 0.5375, data not shown), indicating that all METH-pretreated groups used during the bupropion LTP experiment had similar levels of dopamine neurotoxicity.

### METH impairs LTP in the DMS

**Fig. 2:**
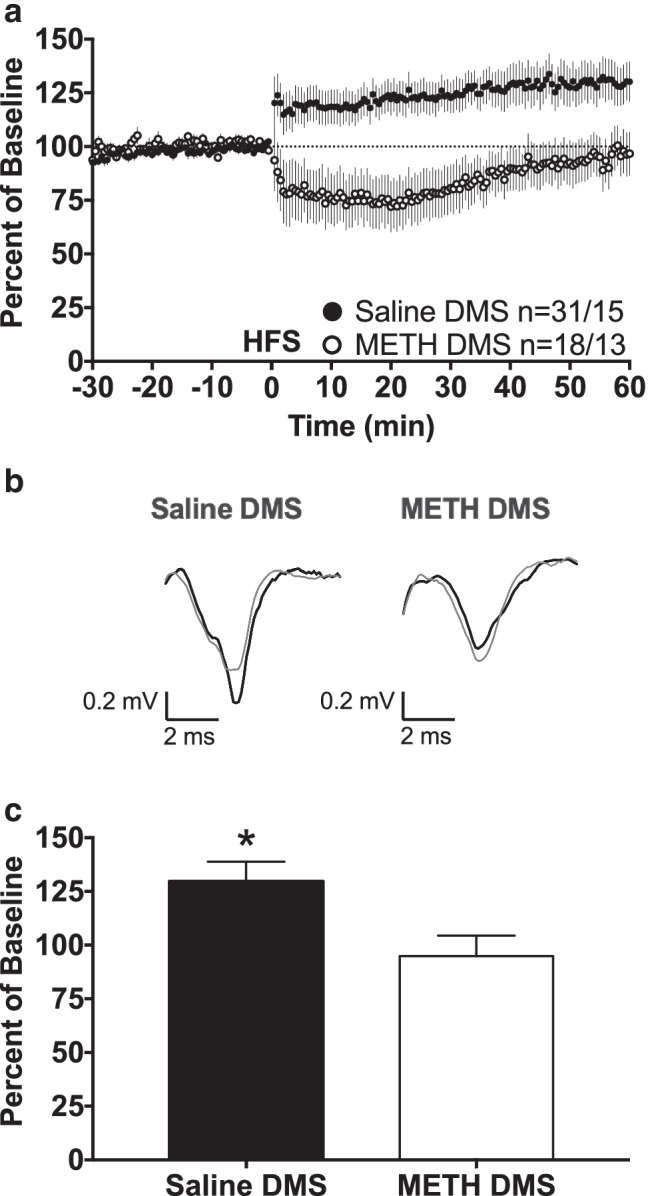
METH impairs LTP in the DMS. **a** fEPSP amplitudes are expressed as a percent of pre-HFS baseline. One second of 100-Hz HFS occurred at time 0. A two-way repeated measures ANOVA of the final 10 min revealed a significant main effect of pretreatment (*F*_(1, 47)_ = 6.226, *p* = 0.0162). Group size is displayed as *n* = slice number/mouse number. **b** Representative fEPSP traces. Thin gray fEPSP traces are pre-HFS traces; thick black fEPSP traces are post-HFS traces. **c** Group average fEPSP amplitude during the final 10 min of recording. Only the Saline DMS group average was significantly higher than 100%, indicating LTP in the Saline DMS group, but not in the METH DMS group. With the exception of **b**, all data are expressed as group mean ± *SEM*. *One-sample *t*-test, significantly different from a theoretical value of 100%, *p* < 0.05

Corticostriatal plasticity is an important feature of striatal function, and impairments in corticostriatal plasticity likely impair broader striatal function. Within the DMS, 1-s HFS induced LTP in the saline-pretreated group, but not in the METH-pretreated group (Fig. 2a). A two-way repeated measures ANOVA of the final 10 min of recording revealed a significant main effect of pretreatment (*F*_(1, 47)_ = 6.226, *p* = 0.0162), demonstrating a significant difference between the saline-pretreated and METH-pretreated groups. In addition, a one-sample *t*-test of the average fEPSP amplitudes for each group during the final 10 min of recording found that the fEPSP amplitude for the saline-pretreated group was significantly different from a theoretical baseline value of 100% (Fig. 2c, Saline DMS: 129.7 ± 9.1, *n* = 31, *t* = 3.267, *df* = 30, *p* = 0.0027), and therefore expressed LTP. In contrast, a one-sample *t*-test found that the average fEPSP amplitude of the METH-pretreated group was not significantly different from a theoretical baseline value of 100% (Fig. 2c, METH DMS: 94.8 ± 9.6, *n* = 18, *t* = 0.546, *df* = 17, *p* = 0.5922), indicating an absence of long-term plasticity in this group. In summary, there was a significant difference in average fEPSP amplitudes during the final 10 min of recording between the saline-pretreated DMS group and METH-pretreated DMS group. The saline-pretreated DMS group expressed LTP, but the METH-pretreated DMS group did not. Thus, the METH binge regimen impairs LTP within the DMS.

### One-second HFS does not elicit long-term plasticity in the DLS

**Fig. 3:**
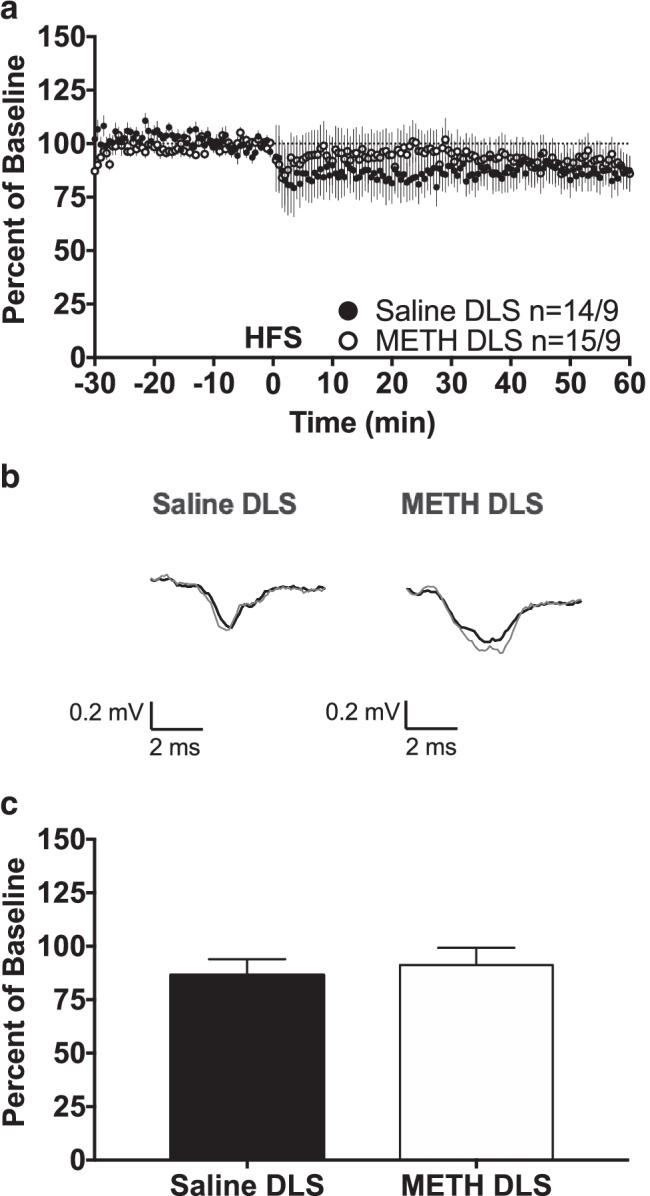
One-second HFS does not elicit long-term plasticity in the DLS. **a** fEPSP amplitudes are expressed as a percent of pre-HFS baseline. One second of 100-Hz HFS occurred at time 0. Group size is displayed as *n* = slice number/mouse number. **b** Representative fEPSP traces. Thin gray fEPSP traces are pre-HFS traces; thick black fEPSP traces are post-HFS traces. **c** Group average fEPSP amplitude during the final 10 min of recording. Neither group average was significantly different from 100%, indicating that neither group exhibited long-term plasticity. With the exception of **b**, all data are expressed as group mean ± *SEM*

In contrast to the DMS, 1-s HFS did not elicit long-term plasticity in the DLS of either saline- or METH-pretreated mice (Fig. 3a). A two-way repeated measures ANOVA of the last 10 min of recording revealed no significant effect of pretreatment (*F*_(1, 27)_ = 0.1763, *p* = 0.6779). In addition, a one-sample *t*-test found that neither the saline- nor the METH-pretreated group was significantly different from a theoretical baseline value of 100% (Fig. 3c, Saline DLS: 86.5 ± 7.5, *n* = 14, *t* = 1.809 *df* = 13, *p* = 0.0936; METH DLS: 91.2 ± 8.1, *n* = 15, *t* = 1.087, *df* = 14, *p* = 0.2955). Therefore, neither of the groups expressed long-term plasticity within the DLS.

### LTP in the DMS is dependent on dopamine D1 receptor activation

**Fig. 4:**
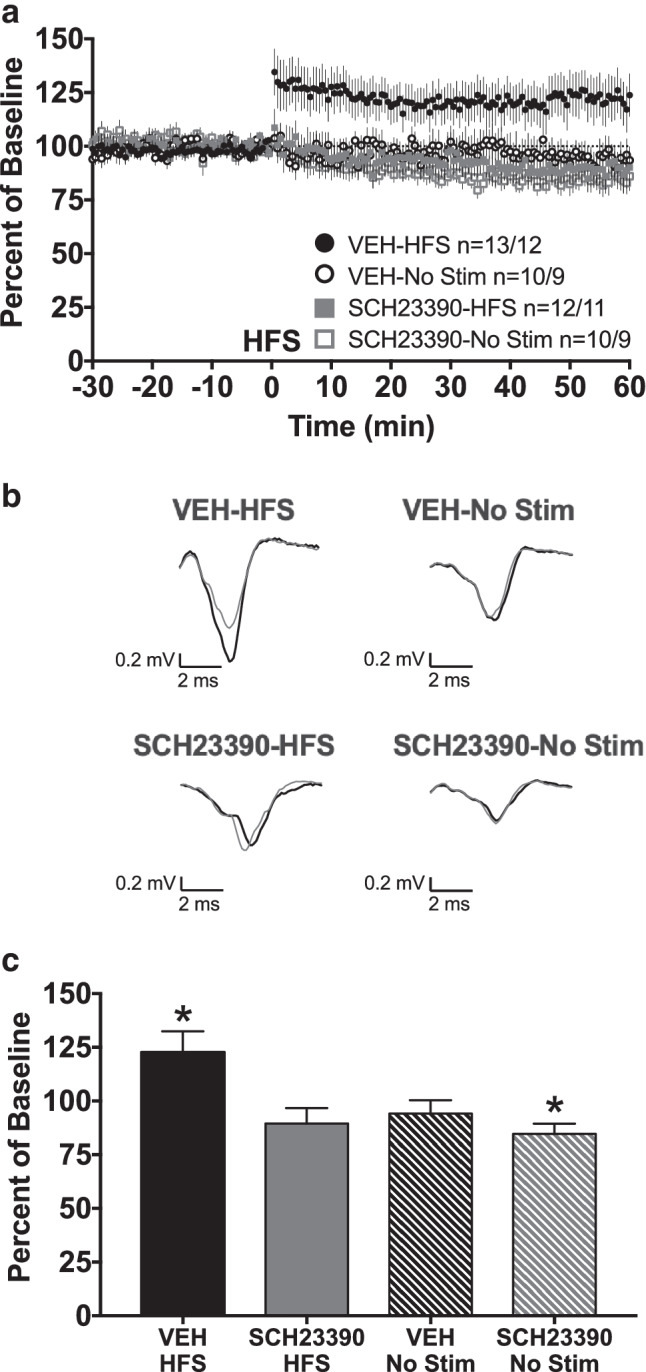
LTP in the DMS is dependent on dopamine D1 receptor activation. **a** fEPSP amplitudes are expressed as a percent of pre-HFS baseline. One second of 100-Hz HFS occurred at time 0. Slices were continuously perfused with either ACSF + 10 μM SCH23390 or ACSF + vehicle throughout the experiment. A repeated measures MANOVA of the final 10 min revealed a significant main effect of drug (*F*_(1, 41)_ = 7.4484, *p* = 0.0093) and significant main effect of stimulation (*F*_(1, 41)_ = 4.5633, *p* = 0.0387). Post hoc Dunnett’s multiple comparison test revealed that all experimental groups were significantly different from the VEH-HFS control group (VEH-HFS vs VEH-No Stim: *q* = 2.61, *df* = 41, *p* = 0.0334; VEH-HFS vs SCH23390-HFS: *q* = 3.187, *df* = 41, *p* = 0.0076; VEH-HFS vs SCH23390-No Stim: *q* = 3.471, *df* = 41, *p* = 0.0035). Group size is displayed as n = slice number/mouse number. **b** Representative fEPSP traces. Thin gray fEPSP traces are pre-HFS traces; thick black fEPSP traces are post-HFS traces. **c** Group average fEPSP amplitude during the final 10 min of recording. Only the VEH-HFS group average was significantly higher than 100%, indicating LTP in the VEH-HFS group; all other groups showed average fEPSP amplitudes less than 100%, suggesting rundown of the fEPSP amplitude over time, with the average in the SCH23390-No Stim group being statistically significantly lower. With the exception of **b**, all data are expressed as group mean ± *SEM*. *One-sample *t*-test, significantly different from a theoretical value of 100%, *p* < 0.05

Dopamine is an important bidirectional modulator of some forms of corticostriatal plasticity (Lovinger 2010), although the role of dopamine signaling in LTP induced by 1-s HFS has not been previously established. In order to determine if LTP induced in the DMS through this stimulation method requires D1 receptor signaling, DMS slices from control mice were continuously perfused with either ACSF containing the dopamine D1 receptor antagonist SCH23390 (10 μM) or vehicle (VEH) and exposed to either 1-s HFS or no stimulation (No Stim). Thus, the four groups were VEH-No Stim, VEH-HFS, SCH23390-No Stim, and SCH23390-HFS. A repeated measures MANOVA on the last 10 min of recording revealed a significant main effect of drug (Fig. 4a, F_(1, 41)_ = 7.4484, *p* = 0.0093) and a significant main effect of stimulation (*F*_(1, 41)_ = 4.5633, *p* = 0.0387). A post hoc Dunnett’s multiple comparison test was used to compare each group average during the final 10 min to the VEH-HFS positive control group. Each group was significantly different from the VEH-HFS control (VEH-HFS vs VEH-No Stim: *q* = 2.61, *df* = 41, *p* = 0.0334; VEH-HFS vs SCH23390-HFS: *q* = 3.187, *df* = 41, *p* = 0.0076; VEH-HFS vs SCH23390-No Stim: *q* = 3.471, *df* = 41, *p* = 0.0035). In addition, a one-sample *t*-test found that the VEH-HFS group was significantly different from a theoretical baseline value of 100% (Fig. 4c, VEH-HFS: 122.6 ± 9.9, *n* = 13, *t* = 2.29, *df* = 12, *p* = 0.0410), indicating LTP within this group. However, the SCH23390-HFS group was not greater than a theoretical baseline value of 100% (Fig. 4c, SCH23390-HFS: 89.5 ± 7.2, *n* = 12, *t* = 1.451, *df* = 11, *p* = 0.1746). In fact, the SCH23390-HFS group was less than, although not statistically significantly so, the theoretical value of 100%. Similarly, the VEH-No Stim group was less than, but not statistically significantly so, a theoretical baseline of 100% (Fig. 4c, VEH-No Stim: 94.1 ± 6.3, *n* = 10, *t* = 0.9395, *df* = 9, *p* = 0.3720), and the SCH23390-No Stim group was also lower than a theoretical baseline value of 100%, in this case reaching statistical significance. (Fig. 4c, SCH23390-No Stim: 84.7 ± 4.7, *n* = 10, *t* = 3.256, *df* = 9, *p* = 0.0099). The decrease in fEPSP amplitude in the latter groups likely simply reflects a rundown of fEPSP amplitude with time. Regardless of this small decrease over time, together, the loss of LTP in the SCH23390-HFS group, but not the VEH-HFS group, demonstrates that the LTP evoked in the DMS through 1-s HFS is dependent on dopamine D1 receptor signaling.

### Bupropion restores induction of LTP in METH DMS slices

Given the demonstrated dopamine toxicity induced by the METH binge regimen, the established METH-induced decrement in phasic dopamine signal amplitude (Howard et al. 2011, 2013; Robinson et al. 2014), and the demonstrated importance of D1 receptor signaling for the expression of LTP, we reasoned that pharmacologically restoring the amplitude of the phasic dopamine signal might restore LTP in METH-pretreated mice. Bupropion, an FDA-approved medication used for smoking cessation and treatment of depression, is known to inhibit dopamine uptake (Stamford et al. 1989), increase dopamine uptake into synaptic vesicles (Rau et al. 2005), and increase electrically evoked phasic-like dopamine signals (May et al. 1988). Therefore, we administered bupropion (BUP 25 mg/kg, BUP 50 mg/kg, or VEH) to METH-pretreated mice 30 min before sacrifice to determine if acute, in vivo bupropion treatment would be sufficient to restore ex vivo LTP. Thus, the three groups were METH-VEH, METH-BUP 25 mg/kg, and METH-BUP 50 mg/kg.

**Fig. 5:**
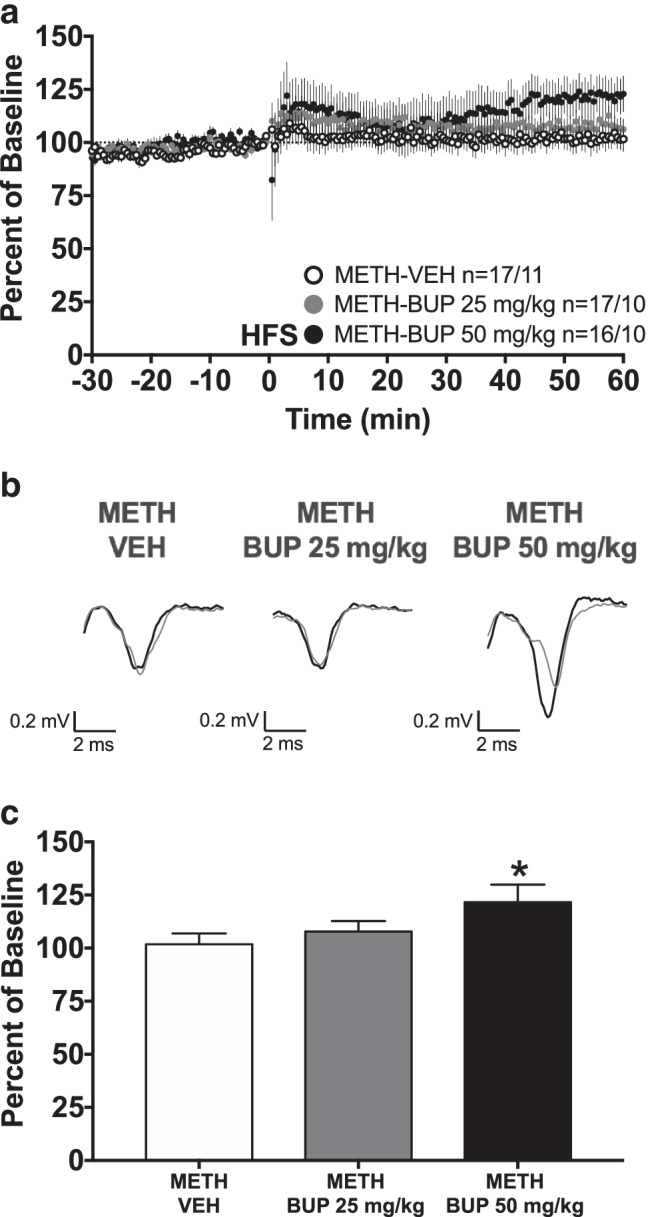
Bupropion restores the induction of LTP in METH DMS slices. **a** fEPSP amplitudes are expressed as a percent of pre-HFS baseline. One second of 100-Hz HFS occurred at time 0. Group size is displayed as *n* = slice number/mouse number. **b** Representative fEPSP traces. Thin gray fEPSP traces are pre-HFS traces; thick black fEPSP traces are post-HFS traces. **c** Group average fEPSP amplitude during the final 10 min of recording. Only the METH DMS BUP 50 mg/kg group average was significantly higher than 100%, indicating that bupropion 50 mg/kg restores the induction of LTP. With the exception of **b**, all data are expressed as group mean ± *SEM*. *One-sample *t*-test, significantly different from a theoretical value of 100%, *p* < 0.05

Within the DMS, a two-way repeated measures ANOVA of the last 10 min of recording found only a non-significant trend toward a main effect of treatment (Fig. 5a, F_(2, 47)_ = 2.684, *p* = 0.0787). However, the one-sample *t*-test found that the average fEPSP of the METH-BUP 50 mg/kg group was significantly different from a theoretical baseline value of 100%, indicating LTP within this group (Fig. 5c, METH-BUP 50 mg/kg: 121.7 ± 8.2, *n* = 16, *t* = 2.631, *df* = 15, *p* = 0.0189). Neither the average fEPSP amplitude of the METH-VEH group nor the METH-BUP 25 mg/kg group was significantly different from a theoretical baseline of 100% (Fig. 5c, METH-VEH: 101.8 ± 5.1, *n* = 17, *t* = 0.3481, *df* = 16, *p* = 0.7323; METH-BUP 25 mg/kg: 107.8 ± 4.9, *n* = 17, *t* = 1.587, *df* = 16, *p* = 0.1320), and therefore did not express long-term plasticity. Thus, while the groups were not significantly different from one another on the two-way repeated measures ANOVA, the METH-BUP 50 mg/kg group did express LTP, whereas the METH-VEH group and METH-BUP 25 mg/kg group did not, suggesting that acute in vivo bupropion treatment (50 mg/kg) 30 min prior to sacrifice restores the induction of LTP in the DMS of METH-pretreated mice.

## Discussion

This study demonstrates that the METH binge regimen damages striatal dopamine terminals and impairs D1 receptor-dependent LTP within the DMS. Furthermore, the FDA-approved drug bupropion, when given in vivo 30 min prior to sacrifice, rescues this deficit, enabling the induction of LTP in mice with METH-induced neurotoxicity.

When assessed approximately 3–5 weeks after exposure to a binge regimen of METH, we found reduced DAT expression in the DMS and DLS of METH-pretreated mice, confirming a lasting, partial dopamine lesion induced by the METH binge regimen. Long-term dopamine toxicity, including dopamine terminal damage (Kogan et al. 1976; Wagner et al. 1980; Grace et al. 2010; Ares-Santos et al. 2014), is a well-established observation following such METH exposure in mice and rats. Therefore, the mice in our studies reliably replicated the known neurotoxic effects of METH exposure on striatal dopamine innervation. The results presented herein, however, extend our knowledge of the long-term impact of METH-induced neurotoxicity, as this is the first study to characterize the effects of this persistent, partial dopamine loss induced by prior exposure to METH on long-term striatal plasticity.

Although the present findings are the first to characterize how METH-induced neurotoxicity affects striatal LTP, there are existing studies on the effects of other METH-exposure paradigms on striatal synaptic plasticity, with some diversity in results. For example, Huang et al. (2017) reported impaired LTD (and a trend toward LTP) in DLS of slices taken from rats 3–9 days into withdrawal from METH self-administration (3 h/day for 10 days). Similarly, a switch from LTD to LTP and enhanced population spike amplitude has been reported in mice given a sensitizing regimen of METH (6 daily injections followed by 7 days of withdrawal) (Moriguchi et al. 2015). Conversely, Bamford et al. (2008) reported “chronic presynaptic depression” of glutamate release in the forelimb motor region of the dorsal striatum of mice treated with a sensitizing regimen of METH. Finally, acute application of METH to a slice preparation also has been reported to inhibit striatal LTP induced by HFS (Avchalumov et al. 2020). Importantly, in these studies, the dopamine innervation was reported to be intact (Bamford et al. 2008) or was not assessed. It is thus evident that METH exposure, either previously or acutely, can effect changes in striatal synaptic plasticity. The present results extend this understanding by providing the first report of the impact of METH-induced dopamine neurotoxicity on striatal plasticity. Clearly, understanding how a history of METH abuse affects striatal plasticity and, consequently, basal ganglia-mediated learning and memory processes will require consideration of the nature of the METH-exposure history, the presence or absence of METH in the system at the time of evaluation, and, in light of the present findings, the impact of any METH-induced neurotoxicity.

Studies of dopamine depletion induced with the neurotoxin 6-OHDA have demonstrated that both full (Centonze et al. 1999; Kerr and Wickens 2001; Picconi et al. 2003; Shen et al. 2008, 2015; Paille et al. 2010; Skiteva et al. 2018) and partial (Paille et al. 2010) dopamine lesions impair striatal LTP. Our work similarly found that mice with METH-induced partial dopamine lesions have impaired LTP. Both full and partial dopamine depletions likely impair LTP due to reduced dopamine D1 receptor signaling and decreased activation of PKA and DARPP-32 (Centonze et al. 2001), as LTP can be restored following partial or full dopamine depletion by administration of a dopamine D1 receptor agonist (Kerr and Wickens 2001; Shen et al. 2008; Paille et al. 2010). In contrast, D1 receptor inhibition, either through D1 receptor knockout or D1 receptor antagonism, impairs striatal LTP (Centonze et al. 2003; Pawlak and Kerr 2008; Shen et al. 2008; Hawes et al. 2013). The D1 receptor antagonist SCH23390 similarly impaired LTP elicited through our induction method of 1-s HFS, further supporting the conclusion that disruption of LTP in mice with METH-induced striatal dopamine neurotoxicity is likely due to disruption of dopamine D1 receptor activation secondary to partial dopamine depletion.

Work by Dreyer et al. (2010) suggests that phasic dopamine signaling primarily activates D1 receptors (but see also Hunger et al. (2020)). A METH-induced reduction in phasic dopamine signaling may therefore result in a reduction in D1 receptor signaling and impairment in D1 receptor-dependent LTP. Because striatal MSNs generally selectively express either D1 or D2 receptors, any LTP deficits arising due to insufficient D1 receptor signaling may be specific to the D1-MSN population. In support of this, LTP is impaired in D1-MSNs but normal in D2-MSNs from mice with full dopamine depletions (Shen et al. 2008; Thiele et al. 2014). In contrast, LTP within D2-MSNs is likely not dopamine-dependent (Shen et al. 2008). While our field potential recordings were not cell-type specific, it is possible that METH impairs LTP within D1-MSNs, but does not affect LTP in D2-MSNs. Future cell-type-specific studies will therefore be needed to determine if the METH-induced LTP deficit observed in this study is restricted to D1-MSNs.

Not surprisingly, the 1-s HFS used in this study did not elicit long-term plasticity in the DLS of either the saline-pretreated or METH-pretreated group. Plasticity induction methods within the striatum vary significantly across different labs and studies; however, across studies, LTP is more prevalent in the DMS than DLS. While the basis underlying this difference is not fully elucidated, differences in dopamine receptor abundance (e.g., greater dopamine D2 receptor expression in DLS), as well as differences in glutamate, acetylcholine, and endocannabinoid signaling as extensively reviewed by Lovinger (2010), likely contribute to this well-documented difference in ability to induce LTP in the DMS versus DLS. In relation to the present findings, other work has similarly found that a stimulation method used to induce LTP in the DMS did not induce any long-term plasticity in the DLS (Hawes et al. 2013). The 1-s HFS protocol used herein has been used previously to successfully induce LTP in the DMS (Nagarajan et al. 2017), and the present results thus provide the first replication of that observation while extending those findings to show for the first time that the 1-s HFS paradigm does not induce LTP or LTD in the DLS. The present study was the first to examine the effects of 1-s HFS in the DLS. Clearly, further work examining mechanisms underlying plasticity in both the DMS and DLS in normal animals, as well as in the setting of METH-induced dopamine neurotoxicity, continues to be necessary to fully understand regional and cellular differences in striatal plasticity.

Bupropion is an FDA-approved drug that has been used in multiple clinical trials for METH use disorder, with varying results (Newton et al. 2006; Elkashef et al. 2008; Shoptaw et al. 2008; Heinzerling et al. 2014; Anderson et al. 2015; Trivedi et al. 2021), suggesting that bupropion may aid with recovery from METH use disorder, but further mechanistic research and clinical trials are needed to optimize outcomes. In our study, acute, in vivo administration of bupropion (50 mg/kg) restored the induction of LTP in ex vivo DMS slices from METH-pretreated mice. Previous studies have similarly found that in vivo treatment with dopaminergic drugs such as L-DOPA (Shen et al. 2015) or the D1 receptor agonist SKF38393 (Paille et al. 2010) restores LTP induced ex vivo in dopamine-depleted striatal slices. Bupropion likely augments dopamine signaling through a combination of mechanisms. Bupropion blocks dopamine reuptake from the synapse (Stamford et al. 1989), increases vesicular dopamine uptake through redistribution and enhancement of VMAT-2 function (Rau et al. 2005), and increases striatal extracellular dopamine concentrations following phasic-like stimulation (May et al. 1988). While the precise effects of bupropion on dopamine signaling were not tested in this study, the known dopaminergic effects of this drug suggest that bupropion-induced augmentation of phasic dopamine signaling restored the induction of D1-dependent LTP evoked in our study, despite the METH-induced partial dopamine lesion.

It is important to note that bupropion is also a nicotinic acetylcholine receptor (nAChR) antagonist (Slemmer et al. 2000), including the striatally expressed α_4_β_2_ nAChR subtype (Slemmer et al. 2000; Quik and Wonnocott 2011). Consequently, it is possible that the effects of bupropion reported herein may be due to bupropion-induced blockade of nAChRs. The α_4_β_2_ nAChR antagonist dihydro-β-erythroidine (DHβE) impairs D2 receptor-dependent LTD within the striatum (Partridge et al. 2002), which could conceivably then unmask LTP. In this regard, previous work has reported that an induction stimulus can activate opposing signaling pathways promoting both striatal LTP and LTD in the same neuron (Shen et al. 2008). It is therefore possible that nAChR antagonism by bupropion impaired LTD signaling pathways, effectively disinhibiting weak LTP expression that may have been otherwise masked within DMS slices from METH-pretreated mice. The present work therefore presents the first evidence that bupropion promotes LTP in mice with METH-induced neurotoxicity, but further work is needed to identify bupropion’s specific mechanism of action in order to inform the development of improved therapeutic approaches to managing METH use disorder.

In summary, this study provides the first evidence that METH-induced neurotoxicity impairs dopamine D1 receptor-dependent, corticostriatal LTP in the DMS. This deficit in LTP is likely due to the partial dopamine loss induced by METH and consequent METH-induced reduction in phasic dopamine signaling through dopamine D1 receptors within the striatum. Given that the amplitude of the phasic dopamine signal is driven by dopamine release (Venton et al. 2003), we treated mice with bupropion, a drug that increases vesicular dopamine uptake and enhances dopamine release in response to phasic-like stimulation. Whereas LTP was not induced by the HFS protocol in DMS slices from METH-pretreated mice given vehicle prior to sacrifice, it was induced in METH-pretreated mice given the highest dose of bupropion. Together, these findings indicate that METH-induced partial loss of striatal dopamine is associated with pronounced disruption to striatal plasticity, which can be pharmacologically addressed by bupropion. Future studies will focus on the effects of METH-induced dopamine loss on synaptic plasticity mechanisms in specific MSN subtypes, as well as on the ability of different pharmacological agents that restore phasic dopamine signaling or block nAChRs to address synaptic, molecular, systems, and behavioral functions of the striatum disrupted by METH-induced neurotoxicity.
